# Short Intussusception Valves Prevent Reflux After Jejunal Interposition Bilioduodenal Anastomosis

**DOI:** 10.1155/1991/70270

**Published:** 1991

**Authors:** Liu Xunyang, Liu Shu, Shen Lirong, Pan Aiyin, Wen Jifang, Zhang Shisui, Han Ming, G. V. Stiegmann

**Affiliations:** 1 The Departments of Surgery and Nuclear Medicine The First Affiliated Hospital Hunan Medical College Changsha China; 2 Department of Pathology Hunan Medical College Changsha China; 3 Department of Surgery (Gastrointestinal/Tumor) University of Colorado Denver USA; 4 University of Colorado Health Sciences Center C-313, 4200 East 9th Ave. Denver CO 80262 USA

## Abstract

Short whole circumference and semi-circumference intussusception valves were created in interposition cholecysto-jejunal-duodenal conduits in pigs to determine which method best prevented gastrointestinal reflux into the biliary tract. Following intravenous injection of 99 mTc-HIDA the time interval for its excretion from the liver and appearance in the duodenum was not different in either whole or semi-circumference valve animals or in controls without valves. After intragastric administration of 99 mTc-DTPA the relative radioactivity of gallbladder contents (reflux) in the cohort without valves was significantly higher than in both cohorts with valves. Animals with semi-circumferential valves in turn had significantly higher levels of nuclide than those with whole circumference valves. Reflux was observed grossly in 100% of animals without valves, in 20% of those with semi-circumference valves, and in no animals with whole circumference valves. This study indicates that both Whole and semi-circumference intussusception valves placed in jejunal biliary conduits allow unimpeded flow of bile into the gastrointestinal tract. Whole circumference valves are more effective for prevention of reflux than semi-circumferential valves.


*HPB Surgery*, 1991, Vol. 5, pp. 29–34
Reprints available directly from the publisher
Photocopying permitted by license only

© 1991 Harwood Academic Publishers GmbH
Printed in the United Kingdom

### SHORT INTUSSUSCEPTION VALVES PREVENT REFLUX AFTER JEJUNAL INTERPOSITION BILIODUODENAL ANASTOMOSIS

LIU XUNYANG, LIU SHU, SHEN LIRONG, PAN AIYIN, WEN JIFANG, ZHANG SHISUI, HAN MING and G.V. STIEGMANN

*From The Departments of Surgery and Nuclear Medicine, The First Affiliated Hospital, Hunan Medical College, and the Department of Pathology, Hunan Medical College, Changsha, Department of Surgery (Gastrointestinal/Tumor) University of Colorado, Denver.*

*(Received 25 April 1991)*

Short whole circumference and semi-circumference intussusception valves were created in interposition cholecysto-jejunal-duodenal conduits in pigs to determine which method best prevented gastrointestinal reflux into the biliary tract. Following intravenous injection of 99 mTc-HIDA the time interval for its excretion from the liver and appearance in the duodenum was not different in either whole or semi-circumference valve animals or in controls without valves. After intragastric administration of 99 mTc-DTPA the relative radioactivity of gallbladder contents (reflux) in the cohort without valves was significantly higher than in both cohorts with valves. Animals with semi-circumferential valves in turn had significantly higher levels of nuclide than those with whole circumference valves. Reflux was observed grossly in 100% of animals without valves, in 20% of those with semi-circumference valves, and in no animals with whole circumference valves. This study indicates that both whole and semi-circumference intussusception valves placed in jejunal biliary conduits allow unimpeded flow of bile into the gastrointestinal tract. Whole circumference valves are more effective for prevention of reflux than semi-circumferential valves.

KEY WORDS: Bile duct, biliary anastomosis, choledochojejunostomy

### INTRODUCTION

Biliary reconstruction is commonly performed using Roux-en-Y-choledochal or hepaticojejunostomy. Such reconstruction effectively prevents reflux of intestinal contents into the biliary tree but eliminates bile from the duodenum and prohibits future access to the hepatic ducts except by percutaneous transhepatic techniques. Jejunal interposition between bile duct and duodenum has the advantage of conveying bile to the duodenum and thus maintaining a relatively normal duodenal milieu in addition to allowing direct endoscopic examination of the biliary system if the need arises[1,2]. This form of reconstruction however, allows free reflux of duodenal contents into the biliary tree which in some circumstances may have adverse sequelae. The purpose of this study was to determine if short (2 cm) intussusception valves created in a jejuno-biliary conduit can effectively prevent reflux of gastric and duodenal contents into the biliary system while allowing free

Address correspondence to: Greg Van Stiegmann, MD, University of Colorado Health Sciences Center, C-313, 4200 East 9th Ave., Denver, CO 80262, USA

drainage of bile into the duodenum and to further assess the efficacy of whole circumference versus semi-circumferential valves for this purpose.

### MATERIALS AND METHODS

Fifteen pigs of either sex weighing from 15–20 kg had general anesthesia induced with ketamine 15–20mg/kg followed by intravenous injection of demerol lmg/kg, phenergan 0.5mg/kg, and gamma hydroxybutyrate 60 mg/kg. The trachea was intubated and anesthesia maintained with ether. Through a right upper paramedian incision the common bile duct was doubly ligated just above the duodenum. Beginning 15 cm distal to the ligament of Treitz, a segment of jejunum 20 cm in length was isolated with its vascular pedicle intact. Continuity of the proximal and distal jejunum was restored by end-to-end anastomosis. The proximal end of the isolated jejunum was closed. A side-to-side anastomosis 2cm in diameter was performed between the gallbladder and the jejunal limb 1.5cm from its closed end. A 2cm in diameter anastomosis was then inserted between the distal open end of the jejunum and the duodenum 8cm below the pylorus.

The animals were divided into 3 groups. Group I — creation of a whole circumference anti-reflux valve. Seven cm from the jejunoduodenostomy the isolated jejunal limb was cleared of its mesentery for a distance of 4 cm and an isoperistaltic intussusception of the whole circumference of the bowel in this cleared area was constructed with 14 seromuscular sutures. (Figure 1) Group II — creation of a semi-circumference valve: The mesenteric border of the isolated segment was not freed. Beginning 7cm from the jejunoduodenostomy an isoperistaltic intussusception of 4cm of the antimesenteric border of the jejunum was made with 14 seromuscular sutures. The mesenteric side of the bowel was not intussuscepted. Group III — no valve was made. Each group consisted of 5 animals.

Flow of bile via the interposed jejunal segments was assessed thirty days postoperatively when animals were anesthetized and a dose of 2mCi of $^{99}$mTc-HIDA (extracted by Kupffer cells of the liver and excreted in bile) was given intravenously via an ear vein[3]. Monitoring excretion of the isotope from the liver into the interposed jejunum, duodenum and gastrointestinal tract was accomplished with a gamma camera. Determinations of nuclide activity and location were made at intervals of 5,10,15, 30 and 60 minutes respectively after isotope injection.

*In vivo* reflux of liquid intestinal contents into the biliary system was assessed ninety days postoperatively. Reoperation was performed and a stomach tube was inserted and 500–1000 uCi of $^{99}$MTC-DTPA in 100ml of normal saline was administered via the stomach tube. This nuclide is not absorbed from the gastrointestinal tract and appearance of the tracer in the gallbladder could only occur by reflux of the substance from the gastrointestinal trace[3]. Two hours later the abdomen was entered through a bilateral subcostal incision. Fluid in the interposed jejunum above and below the valves was aspirated separately. Isovolumetric radioactivity analysis of the aspirated materials was made with a gamma counter. The relative radioactivity of gallbladder bile was determined by the formula: Radioactivity count per unit volume of bile/radioactivity count per unit volume of intestinal juice $\times$ 100 = relative radioactivity value of bile.

Subsequently, animals were humanely sacrificed and the interposed jejunum was opened throughout its length and its contents were grossly inspected for the

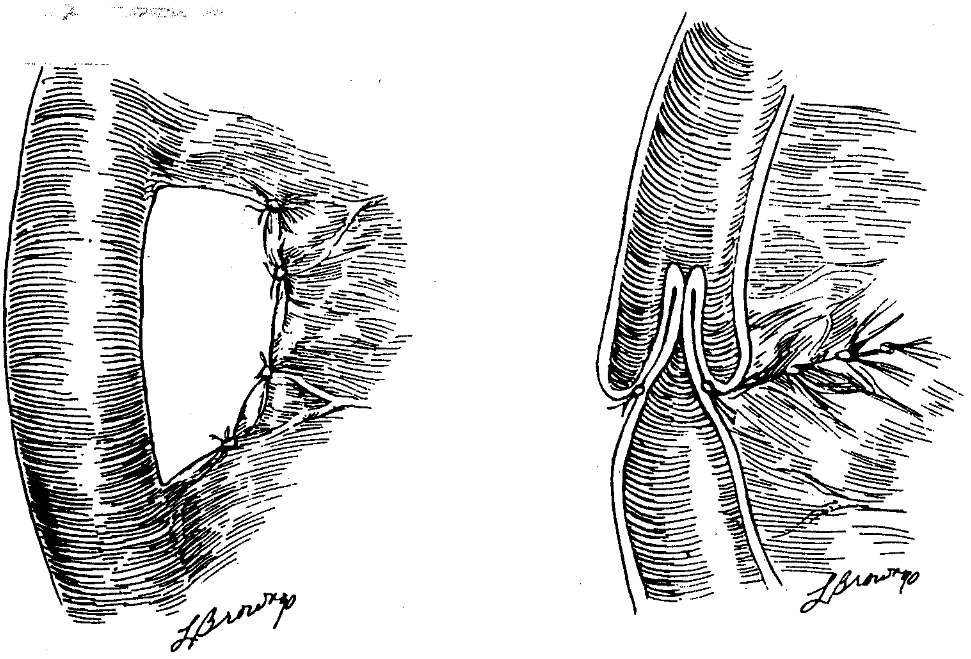

**Figure 1** Creation of a 2cm full circumference anitreflux valve. Left: A four cm segment of mesentery is cleared adjacent to the bowel. Right: The valve is created by intussuscepting the bowel and is secured with multiple interrupted seromuscular sutures.

presence and distribution of chyme and food particles. The length of the valve was measured, the integrity of the sutures determined and the valve was excised and fixed in formalin and sections obtained for light microscopy.

Statistical analysis was by Students t-test for paired variables. Significant differences were considered to exist if P values were less than 0.05.

### RESULTS

All animals survived the initial operation. None exhibited evidence of biliary dysfunction and all continued to gain weight appropriately. Following the intravenous injection of $^{99}$mTc-HIDA the time interval for its excretion from the liver into the jejunal conduit and subsequent passage into the proximal small intestine did not differ significantly in any cohort (Tables 1 and 2).

After the administration of $^{99}$mTc-DTPA the relative radioactivity value of gallbladder contents showed that reflux of nuclide was significantly greater in group 3 (no valve) than in either Group 1 or 2. Similarly, animals in Group 2 (semicircumferential valves) had significantly greater nuclide activity than did those in Group 1 (Table 3).

Gross inspection of the interposed jejunum revealed no evidence of reflux of gastrointestinal contents in any animals in Group I. Reflux was observed in 1

**Table 1** Time interval for 99mTc-HIDA to appear in interposedc jejunum. (Group 1 versus 3: $p > 0.2$, Group 2 versus 3: $p > 0.2$)

| *Group* | *Time (Minutes)* |
|---|---|
| 1 | 7.40 ± 1.58 |
| 2 | 10.50 ± 2.10 |
| 3 | 9.20 ± 2.04 |

**Table 2** Relative radioactivity of gallbladder contents after instillation of 99mTc-DTPA. (Group 1 versus 2: $p < 0.05$, Group 1 versus 3: $p < 0.05$)

| *Group* | *Rel. Radioactivity* |
|---|---|
| 1 | 0.08% ± 0.04 |
| 2 | 5.17% ± 4.94 |
| 3 | 44.56% ± 8.64 |

**Table 3** Relative radioactivity of gallbladder contents after instillation of 99mTc-DTPA. (Group 1 versus 2: $P < 0.05$, Group 1 versus 3: $P < 0.05$)

| Group | Rel. Radioactivity |
|---|---|
| 1 | 0.08% ± 0.04 |
| 2 | 5.17% ± 4.94 |
| 3 | 44.56% ± 8.64 |

animal in Group II and in all 5 pigs in Group III. One pig in the latter group had a small gallbladder calculus.

The length of the whole circumference valve was found to be 1.79 ± 0.09cm, and that of the semi-circumference valve was 1.81 ± 0.09cm. The orifices of the whole circumference valves measured 0.5–1cm in diameter, while those of the semi-circular valves were 0.8–1.2cm in diameter.

Examination of the valves showed the mucosa to be uniformly smooth and pink in color with no superficial ulcers or scarring. The valves were flexible and their orifices protruded slightly like a nipple. Histological examination revealed hypertrophy of the smooth muscle layers of the intestine at the sites of the valves. Hypertrophy was particularly prominent at the lead of the intussusception. The mucosa of the valves was invaded by varying numbers of plasma cells and lymphocytes and some epithelial proliferation was noted. The interposed jejunum without a valve showed extensive chronic inflammation and proliferation with thickening of the epithelium.

### DISCUSSION

Intestinal valves have been constructed to prevent reflux or prolong transit time in the GI tract[4–7]. The ideal valve for bilioduodenal anastomosis with jejunal interposition should prevent reflux, allow the free flow of bile and be easy to construct. This study showed that the intussusception valves did not slow the drainage of bile through the interposed jejunum. This study also showed that all the pigs without intussusception valves had gross reflux of chyme and food debris as well as significantly greater *in vivo* reflux of liquid nuclide tracer as determined by $^{99}$mTc-DPTA studies.

The short intussusception valves created in this model appear effective against reflux of both solid and liquid substances. However, the relative radioactivity value of gallbladder bile was significantly greater for Group 2 than for Group 1 animals suggesting that the whole circumference valve is superior to the semi-circumference valve for prevention reflux of liquids. A possible reason for this is the size of the valve orifice which was 0.5–1cm and 0.8–1.2cm in diameter respectively for the whole circumference valve and the semi-circumference valve. The semi-circumference valve is an incomplete valve, especially when the mesenteric border is wide. The mesenteric border of the jejunum at the site of the valve is not intussuscepted, but the mesentery of this area of bowel lies between two layers of intestinal wall forming a mass which may interfere with the function of the semi-circumference valve for prevention of reflux.

Svensson *et al.*[4,5] reported interposition of an isoperistaltic jejunal segment with an intussusception valve between the stomach and duodenum following partial gastrectomy and found the valve was successful in preventing reflux but observed delayed peristalsis and emptying of the jejunal segment. Their valve was made by intussusception of 8cm of jejunum, making a valve 4 cm long. In our experiments the valves created were only 2cm long. Our shorter valve length may explain why no delayed emptying of the interposed jejunum occurred in this study.

In order to maintain proper position of the valves, Svensson[4] used electrocautery to make numerous transverse incisions in the seromuscular layer of the intestinal segment to be intussuscepted, and constructed the intussusception valve by sutures passed through the mesenteric defect so as to rotate the walls of the intestine around the valve. Our experiments show that these procedures are not necessary for making an effective valve of 2cm in length. All the valves constructed by our simple method retained their length and position and were functionally effective at the conclusion of the study.

### SUMMARY

This study has shown that in a porcine model an intussusception valve 2cm in length constructed in the interposed jejunum used for bilioduodenal anastomosis is effective in preventing reflux and allows the free exit of bile into the gastrointestinal tract. Whole-circumference valves appear more effective at preventing reflux than semi-circumferential valves. This difference may be explained by the smaller effective diameter of the former. Short intussusception valves may be useful clinically when subsequent direct endoscopic access and prevention of intestinal reflux into the biliary tract is desired.

### *References*

1. Wheeler, E.S. and Longmire, W.P. (1978) Repair of benign stricture of the common bile duct by jejunal interposition choledochoduodenostomy. *Surg. Gynecol. Obstet.*, **146**, 260–2
2. Stiegmann, G.V., Mansour, M.A., Geoff, J.S. and Pearlman, N.W. Roux-en-Y jejunoduodenostomy for endoscopic access to hepaticojejunostomy. *Surg. Gynecol. Obstet.*, (in press)
3. Loberg, M.D., Copper, M. and Harvey, E. *et al.* (1976) Development of new radiopharmaceuticals based on N-substitution of iminodiacetic acid. *J.Nucl.Med.*, **17**, 633–8
4. Svensson, J.O. and Kock, N.G. (1980) Intestinal intussusception valve for the prevention of duodenogastric reflux after partial gastrectomy: an experimental study in dogs. *Acta. Chir. Scand.*, **146**, 511–518
5. Svensson, J.O. and Olbe, L. (1985) Intestinal intussusception valve for postgastrectomy bile reflux and/or dumping *Act. Chir. Scand.*, **151**, 355–360
6. Wiklund, B. and Hallberg, D. (1985) Experiences with anti-reflux valves in jejunoileal bypass surgery. *Acta. Chir. Scand.*, **151**, 159–162
7. Ricotta, J., Zuidema, G.D., Gadacz, T.R. and Sadri, D.R. (1981) Construction of iliocecal valve and its role in massive resection of the small intestine. *Surg. Gyn. Obstet.*, **152**, 310–314

*(Accepted by John L. Cameron 2 April 1991)*

